# The Cost of Victory over Cancer: Psychosocial Dysfunction and Depressive Symptoms Among Polish Adolescent Cancer Survivors in the Context of Quality of Life and Psychosocial Health

**DOI:** 10.3390/cancers17243916

**Published:** 2025-12-07

**Authors:** Piotr Pawłowski, Karolina Joanna Ziętara, Joanna Milanowska, Anna Aftyka, Mateusz Sobierajski, Zuzanna Kania, Marzena Samardakiewicz

**Affiliations:** 1 Department of Psychology, Psychosocial Aspects of Medicine, Medical University of Lublin, 20-093 Lublin, Poland; 2 Institute of Medical Sciences, University of Applied Sciences in Chełm, 22-100 Chełm, Poland; 3 Pomeranian Hospitals, 84-200 Wejherowo, Poland; 4 Department of Anaesthesiological and Intensive Care Nursing, Medical University of Lublin, 20-081 Lublin, Poland; 5 Student Scientific Club at the Department of Psychology, Medical University of Lublin, 20-093 Lublin, Poland

**Keywords:** adolescent cancer survivors, health-related quality of life, depressive symptoms, self-esteem, interpersonal functioning, psychosocial adaptation

## Abstract

Background: Adolescent cancer survivors constitute a clinically vulnerable population whose psychosocial adjustment following oncological treatment remains insufficiently characterized, particularly within Central and Eastern Europe. The present study aimed to evaluate health-related quality of life (HRQoL) and depressive symptomatology among Polish adolescent survivors, identify their psychological predictors, and determine age-related differences in these associations. Methods: A cross-sectional study was conducted among 165 survivors aged 11–18 years, recruited from four pediatric oncology centers. Participants completed the KIDSCREEN-10 (HRQoL) and the Children’s Depression Inventory-2™ (CDI-2™). Descriptive statistics, Spearman rank-order correlations, and multiple regression analyses were performed separately for younger (primary school) and older (secondary school) cohorts. Results: The findings demonstrated a pronounced polarization of HRQoL, with approximately one-third of participants (32.7%) scoring within the clinically low range. Depressive symptoms were prevalent, particularly in the domains of Negative Mood (M = 19.93) and Ineffectiveness (M = 15.45), while Negative Self-Esteem levels were comparatively low (M = 8.02). HRQoL correlated strongly and inversely with Interpersonal Problems (rs = −0.89, *p* < 0.001). Regression analyses indicated that Negative Self-Esteem (CDI-2D) was the strongest negative predictor of HRQoL in both age groups, whereas Ineffectiveness (CDI-2C) and Negative Mood (CDI-2A) emerged as significant positive predictors. Interpersonal Problems (CDI-2B) were predictive only in older adolescents, suggesting a developmental shift in determinants of well-being. Conclusions: Adolescent cancer survivors exhibit a distinctive psychological pattern characterized by pronounced emotional distress without pervasive self-devaluation. HRQoL appears highly polarized and primarily determined by self-esteem and interpersonal functioning. These findings underscore the necessity of developmentally tailored psychosocial interventions addressing self-worth, peer reintegration, and socio-economic stressors in survivorship care.

## Simple Summary

This study examined the mental health and quality of life of Polish teenagers who survived cancer. The results show that while many young survivors function well, about one-third experience very low well-being and emotional distress. Most report sadness and a sense of inefficiency, but not a negative view of themselves. The strongest factor lowering quality of life was low self-esteem. For older adolescents, problems with peers also became very important. These findings indicate that young survivors frequently experience emotional and social challenges even after completing treatment. Support programs should focus not only on mental health but also on helping them rebuild confidence, friendships, and everyday life.

## 1. Introduction

The dynamic progress in pediatric oncology, driven by therapeutic innovations, has led to a fundamental paradigm shift from treatment focused solely on saving lives to optimizing long-term survival while maintaining the highest possible quality of functioning. It is estimated that globally, more than 80% of children and adolescents diagnosed with cancer achieve recovery [1,2,3]. As a result, the population of childhood cancer survivors (CCS), particularly adolescents, has been steadily increasing, forming a new clinical group whose needs extend beyond traditional medicine [4].

Adolescence is a critical developmental stage characterized by intensive identity formation, the establishment of peer relationships, and the attainment of psychosocial autonomy. Among adolescent cancer survivors, this process is complicated by a range of specific challenges classified as late effects of treatment [5,6]. These effects are not limited to somatic complications (e.g., cardiologic and endocrine), but also encompass a complex spectrum of psychosocial and emotional difficulties. The most salient include chronic fear of cancer recurrence (FCR), feelings of stigma and “otherness,” challenges in social adaptation, and burdens related to ongoing medical surveillance [7]. The heightened vulnerability to mood disorders, including depression, constitutes a significant burden for the mental health of this group. Such disturbances, often masked by the somatic sequelae of disease and treatment, may lead to social withdrawal, reduced self-esteem, and academic difficulties, directly contributing to decreased health-related quality of life (HRQoL) [8,9].

In Poland, the issue of long-term follow-up (LTFU) care for childhood cancer survivors has gained increasing clinical relevance. Despite progress in the organization of oncological care, systems of psychological support and surveillance remain frequently non-standardized and fragmented [10]. There is a lack of standardized, prospective national studies systematically assessing the psychosocial health status of survivors. Existing data focus primarily on survival rates and somatic complications, leaving a substantial gap in understanding the subjective experiences and mental health of young survivors.

The main objective of the present study is to evaluate the interrelations between health-related quality of life and the severity of depressive symptoms among adolescent survivors of childhood cancer. To achieve this, a cross-sectional diagnostic survey design was employed to identify key psychological factors that serve as predictors of these variables. The results are intended to provide an empirical foundation for designing targeted psychosocial interventions within national survivorship care programs.

## 2. Materials and Methods

### 2.1. Study Design and Procedure

Ethical approval was obtained from the Bioethics Committee of the Medical University of Lublin (approval no. KE-0254/119/06/2024). All research procedures adhered to the principles of anonymity and voluntary participation.

Participants were recruited during routine follow-up visits at four pediatric onco-hematology centers in Poland: the Department of Pediatric Hematology, Oncology and Transplantology at the University Clinical Hospital in Lublin; the “Cape of Hope” Department of Pediatric Oncology and Hematology in Wrocław; the Department of Pediatrics, Hematology and Oncology at the Medical University of Gdańsk; and the Department of Pediatric Oncology and Hematology at the Jagiellonian University Medical College in Kraków. The study was conducted between January and July 2025.

### 2.2. Study Phases

The data collection process was divided into four main phases. In the first phase, identification of potential participants was carried out by members of the research team in collaboration with attending physicians, who screened patients meeting the inclusion criteria and verified the presence of any exclusion criteria.

The second phase involved the information and consent procedure. In accordance with ethical and legal requirements, parents or legal guardians received detailed information about the study’s objectives, procedures, and confidentiality measures, and were asked to provide written informed consent. The same information was presented to adolescents in an age-appropriate manner, and their written assent/consent to participate was also obtained.

During the third phase, questionnaire completion, participants filled out a set of instruments (KIDSCREEN-10, CDI 2™, and an author-designed questionnaire) in a quiet, separate room using mobile tablet devices. The average completion time was approximately 15 min. A trained research assistant was present to answer questions when needed, but did not interfere with the content of the responses.

Finally, in the data quality control phase, the collected data were coded and entered into the statistical analysis database. Identification numbers were used to ensure anonymity and to minimize data entry errors.

### 2.3. Inclusion and Exclusion Criteria

#### 2.3.1. Inclusion Criteria

Participants were considered eligible if they had a confirmed diagnosis of childhood cancer before the age of 18 and had completed active oncological treatment (chemotherapy, radiotherapy, hematopoietic stem cell/bone-marrow transplantation, or curative-intent surgical resection). There was no clinically documented progression or recurrence during the 5 years preceding assessment, and the current clinical status was disease-free. The target age at the time of questionnaire completion ranged from 11 years 0 months 0 days to 18 years 11 months 30 days, which matches the validated range for KIDSCREEN-10 and CDI 2™. Eligible adolescents were required to understand and complete Polish self-report measures; no severe cognitive impairment that would prevent correct comprehension could be present. Ethical and legal requirements included written informed consent from a parent or legal guardian and from the respondent (assent/consent as appropriate), with current residence and care in Poland or close monitoring within a Polish healthcare facility.

#### 2.3.2. Exclusion Criteria

These exclusion criteria were applied to minimize confounding factors, ensuring that the assessed psychosocial dysfunction reflects the long-term sequelae of cancer and its treatment rather than acute, unrelated situational crises. Furthermore, as the study protocol did not include a clinical psychiatrist on the research team, excluding patients with acute, severe psychiatric instability was a necessary ethical measure to ensure patient safety.

Adolescents were excluded if they were undergoing active cancer therapy, maintenance treatment, or intensive management of complications; if a relapse or a second malignant neoplasm had been diagnosed before assessment; or if serious somatic complications (e.g., severe organ failure, acute neurological injury) required hospitalization or intensive care and could preclude reliable questionnaire completion or substantially distort perceived quality of life. Exclusion also applied to current, documented severe psychotic disorder (e.g., schizophrenia), autism spectrum disorder, or intellectual disability (IQ < 70) likely to affect the validity of self-report; ongoing outpatient psychotherapy or psychiatric hospitalization due to a major depressive or anxiety episode within the last 6 months (as a potential confounder); and insufficient proficiency in Polish that would not allow independent and full understanding of the questionnaires. Additional confounding circumstances comprised critical life events: a major traumatic loss (e.g., death of a parent, sibling, close caregiver, or other emotionally significant person) within 12 months; a serious accident or injury (such as traffic accident, injury leading to permanent disability, or physical/sexual violence) requiring medical intervention within 12 months; or a severe family crisis (e.g., sudden parental divorce, loss of housing or livelihood) within 6 months. Finally, refusal to sign assent/consent by the patient or legal guardian, or incomplete questionnaire data (e.g., omission of ≥20% of items in KIDSCREEN-10 or CDI 2™), rendered the case ineligible for reliable statistical analysis.

### 2.4. Research Instruments

In the study procedure, three research instruments were applied—two standardized tools (KIDSCREEN-10 and CDI 2™) and one author-developed questionnaire.

#### 2.4.1. Health-Related Quality of Life (KIDSCREEN-10)

To assess HRQoL, the KIDSCREEN-10 Index was used. The instrument consists of ten standardized items covering physical, psychological, and social dimensions of well-being, which generate the total T-score. Additionally, the study protocol included the standard general health question (‘In general, how would you say your health is?’). Although this item is not part of the psychometric index calculation, it was included in the analysis as a separate, independent variable labeled ‘Self-rated Health’. The last item refers to the participant’s self-perception of general health. The raw score is converted into a standardized T-score (mean = 50, SD = 10), where higher T values indicate better quality of life. The KIDSCREEN-10 questionnaire demonstrates high internal reliability (Cronbach’s α = 0.82 in population studies) and has been validated for use in the Polish adolescent population [11].

#### 2.4.2. Depressive Symptoms (CDI 2™)

To assess the severity of depressive symptoms, the Polish version of the Children’s Depression Inventory 2™ (CDI 2™) youth self-report form was used. This is a widely accepted, standardized instrument for measuring emotional distress in children and adolescents. The questionnaire consists of 28 items, and each item includes three statements describing different levels of symptom intensity (for example, guilt feelings, sadness, or suicidal thoughts). Responses are scored on a 0–1–2 scale, where higher scores indicate greater intensity of depressive symptoms.

The CDI 2™ provides a Total Depression Score as well as results on four subscales: (A) Negative Mood/Somatic Symptoms, (B) Low Self-Esteem, (C) Ineffectiveness, and (D) Interpersonal Problems. The combined score of the first two subscales (A + B) reflects the Emotional Problems index, whereas the sum of the next two (C + D) represents Functional Problems. Raw scores are converted to standardized T-scores and percentiles, which allow for comparison with age- and gender-specific norms within the national population. Higher T-scores indicate greater severity of depressive symptoms and are essential for identifying potential clinical risk among the studied group of cancer survivors.

#### 2.4.3. Sociodemographic and Clinical Characteristics (Author-Designed Questionnaire)

An author-developed questionnaire consisting of ten items was designed to collect contextual and clinically relevant data specific to the study group. The questionnaire included sections addressing: (1) clinical details (type of cancer, time since completion of treatment, and type of complications), (2) psychosocial factors (perceived family and peer support, school satisfaction), and (3) survivor-specific challenges, such as FCR and feelings of stigmatization. The variables derived from the author-designed questionnaire were used as control and predictor variables in regression analyses. To ensure accurate characterization of the study group and to control for potential confounding variables, the first section of the Author Questionnaire served as a demographic sheet. The collected data were divided into two main categories. The first, sociodemographic variables, included chronological age (in full years), gender (male, female), place of residence (rural area, small, medium, or large city), parental education level (mother and father) used as an indicator of socioeconomic status, and current school status (grade and type of school). The second, clinical (oncological) variables, encompassed primary diagnosis (type of neoplasm according to the international ICD-10 classification or study protocol, e.g., ALL, solid tumor), age at diagnosis, time since the end of active treatment (in full years), type of treatment received (chemotherapy, radiotherapy, bone marrow/stem cell transplantation, surgery) and its intensity (categorical variable), as well as the presence of late effects of treatment (binary variable: yes/no) or comorbidities reported by the participant or their caregiver.

These data were used not only for descriptive purposes (Table 1) but also in advanced statistical analyses, such as regression, to determine whether clinical variables acted as predictors or moderators of the relationship between quality of life and depressive symptoms.

### 2.5. Statistical Analysis

Statistical analyses were performed using IBM SPSS Statistics software, version 28.0. The level of statistical significance was set at α = 0.05. Preliminary analyses included tests of normality (Shapiro–Wilk test) and homogeneity of variance (Levene’s test) for continuous variables, in order to verify assumptions for parametric testing. To examine the research hypotheses concerning the strength and direction of relationships between demographic, clinical, and psychometric variables (KIDSCREEN-10 and CDI 2™ scores), the Spearman rank correlation coefficient (rs) was applied. The choice of a non-parametric test was determined by the distribution characteristics of the analyzed variables, as confirmed by the normality tests. Next, to identify the key predictors of health-related quality of life, multiple linear regression models were conducted. This analysis aimed to determine the extent to which depressive symptoms (CDI 2™ subscales) and other factors (e.g., age) served as independent predictors of the overall quality of life index (total KIDSCREEN-10 score).

To account for developmental differences, regression analyses were performed separately for two subgroups defined by the participants’ current stage of education: the Younger Cohort (students attending primary school; ages approx. 11–15) and the Older Cohort (students attending secondary school; ages approx. 15–18). This stratification reflects the structural threshold in the Polish education system, which marks a significant transition in the adolescent social environment.

## 3. Results

### 3.1. Characteristics of the Study Group

A total of 170 adolescents participated in the study. Following data verification, five respondents were excluded due to missing data exceeding 20%, which made their inclusion in further analyses impossible. The qualified study sample was divided into two subgroups according to their current stage of education: the Younger Cohort (primary school; n = 89) and the Older Cohort (secondary school; n = 76). All demographic variables are presented in Table 1.

The examined sample showed an almost balanced gender distribution and a comparable proportion of students from primary and secondary education levels, with the most common age being 13 years (21.21%). The participants’ family background appeared largely stable: the majority of respondents came from two-parent families (80.00%) and lived with both parents (80.61%). A high level of parental education was also observed, with higher or secondary education predominating among both mothers and fathers, jointly exceeding 80% in each group.

### 3.2. Results of Psychological Questionnaires (KIDSCREEN-10 and CDI-2™)

Psychometric analysis confirmed the high reliability of both instruments used in the study sample (N = 165). For the KIDSCREEN-10 Index, internal consistency was satisfactory, with a Cronbach’s alpha coefficient of 0.84. This result is highly consistent with the reference values reported in European population studies (Cronbach’s α = 0.82) [11]. Similarly, the CDI-2™ demonstrated strong internal consistency: the Cronbach’s alpha for the Total Depression Score was 0.89, which aligns with the reliability standards reported in the validation studies of the tool (α > 0.80). These indicators confirm the psychometric validity of the measurements in the studied cohort.

The KIDSCREEN-10 results indicate a moderately high yet highly diversified level of well-being within the study group (N = 165). The arithmetic mean (M = 35.93) and median (Mdn = 37.00) show a concentration of scores within the middle-to-upper range of the scale. The distribution is bimodal, with two dominant values at 38 and 39 (each N = 18). This pattern suggests the existence of two distinct subgroups differing in perceived quality of life. Descriptive statistics for the KIDSCREEN-10 are presented in Table 2.

Such polarization is further reflected in the percentile analysis for the Polish population, presented in Table 3. The categorization of HRQoL levels (Table 3) was established based on the standardized T-score distribution for the Polish adolescent population (Mean = 50, SD = 10), as defined in the KIDSCREEN manual [12]. Clinical risk categories were operationalized using standard deviation cut-points: scores falling below 1 SD from the population mean (T < 40) indicate ‘Low Well-being,’ while scores below 2 SD (T < 30) indicate ‘Critical/Very Low Well-being,’ warranting immediate clinical attention. This stratification allows for the identification of survivors who deviate significantly from peer norms. The dominant percentile category falls within the lowest range (<10th percentile), representing 32.73% of the valid sample (N = 54). This means that almost one-third of respondents report a subjectively very low quality of life. Nevertheless, the majority of participants (68.23%) fall within the higher percentile categories.

Regarding depressive symptoms, the CDI-2™ results demonstrate high homogeneity and symmetry, suggesting a uniform level of reported difficulties (total score range: 18–60). The measures of central tendency were nearly identical (M = 56.06, Mdn = 56.00). The distribution was highly symmetrical, close to normal, with a clear mode (D) at 56 (N = 30). Extremely high intensity of Negative Mood symptoms was observed (M = 19.93), very close to the maximum possible score of 21, with a mode of 21, suggesting widespread and strong experiences of dysphoria and sadness. In the Ineffectiveness subscale, results were also elevated (M = 15.45), indicating a considerable sense of inefficacy and low agency. Interpersonal Problems were found to be within a moderate range of difficulty (M = 13.26). Negative Self-Esteem was the only area with a low intensity of symptoms (M = 8.02), suggesting that respondents, despite strong mood symptoms, do not intensely internalize negative self-image. Descriptive statistics for the CDI-2™ are shown in Table 4.

Overall, the analysis reveals a discrepancy in the psychological profile of the sample. On one hand, there is an extremely high intensity of negative mood (CDI-2 subscale A) and inefficacy (CDI-2 subscale C); on the other, a markedly heterogeneous distribution of well-being (KIDSCREEN-10). This contrast is statistically evident when comparing the heterogeneity of the results: the KIDSCREEN-10 scores demonstrate a higher standard deviation (SD = 5.17), indicating greater diversity in perceived quality of life, whereas the CDI-2™ scale shows high homogeneity (SD = 4.20), reflecting a more uniform experience of emotional distress across the group.

### 3.3. Analysis of Relationships Between Quality of Life (KIDSCREEN-10) and Depressive Symptoms (CDI-2™): Spearman Rank Correlations

The strongest relationship identified in the study was a very strong negative correlation between the overall KIDSCREEN-10 quality of life score and the CDI-2™ subscale D (Negative Self-Esteem) (rs = −0.89, *p* < 0.001). This indicates that subjective well-being is most closely associated with negative self-evaluation. Significant positive correlations of moderate to strong magnitude were also identified between the total KIDSCREEN-10 score and specific CDI-2™ domains. A strong correlation was observed with Subscale A (Negative Mood/Somatic Symptoms; rs = 0.69), and a moderate correlation with Subscale B (Interpersonal Problems; rs = 0.49). Additionally, global quality of life correlated strongly with the overall Emotional Problems index (rs = 0.71) and moderately with the Functional Problems index (rs = 0.30). The positive direction of these correlations results from the reverse scoring structure of the CDI-2™ for these specific scales (where higher scores in the correlation matrix reflect better functioning in the context of quality of life analysis). Analysis of internal relationships within the CDI-2™ scales revealed that Subscale A (Negative Mood/Somatic Symptoms) was strongly correlated with the overall Emotional Problems index (rs = 0.87). Similarly, Subscale B (Interpersonal Problems) showed a strong correlation with Emotional Problems (rs = 0.80). Age was found to be moderately and negatively correlated with Subscale A (rs = −0.31), suggesting that older participants reported slightly lower intensity of mood symptoms compared to younger adolescents. Furthermore, a moderate negative correlation was observed between Subscale B (Interpersonal Problems) and the number of years since treatment completion (rs = −0.28), indicating that a longer time since the end of therapy is associated with fewer reported interpersonal difficulties. Spearman’s Rank Corralation Matrix is shown in the Table 5.

### 3.4. Predictors of Quality of Life (KIDSCREEN-10) in Younger (M) and Older (S) Children: Multiple Regression Analysis

To identify the key predictors of the dependent variable, the overall quality of life (total KIDSCREEN-10 score), multiple linear regression models were applied (Table 6). The analysis was conducted separately for the Younger Cohort (primary school students) and the Older Cohort (secondary school students). For both models, the following independent variables (predictors) were entered: age, self-assessed health status, and the four symptomatic subscales of the CDI-2™: (A) Negative Mood/Somatic Symptoms, (B) Low Self-Esteem, (C) Ineffectiveness, and (D) Interpersonal Problems.

Prior to interpreting the regression coefficients, collinearity diagnostics were performed to ensure the stability of the models. The Variance Inflation Factor (VIF) was calculated for all predictors. In both the younger and older cohorts, VIF values were low, ranging from 1.15 to 2.43 (well below the commonly accepted threshold of 5). This confirms that multicollinearity did not distort the results and that the high R2 values reflect genuine predictive power rather than statistical redundancy.

**Figure 1:**
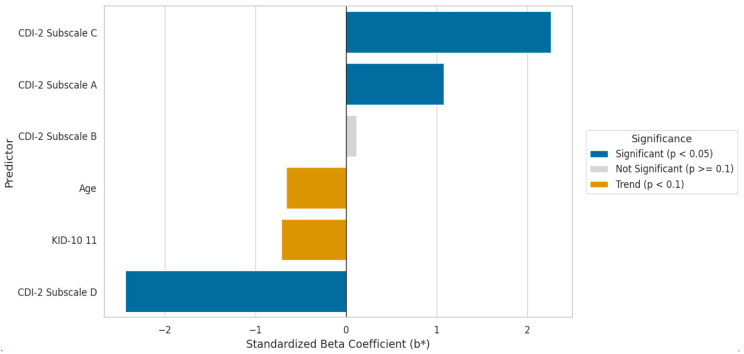
Predictors of Quality of Life—Younger Cohort (N = 89).

The regression model for the Younger Group showed a very high level of fit to the empirical data (R = 0.959). The predictors together explained 91.4% of the variance of the dependent variable (adjusted R^2^ = 0.9136), indicating strong predictive power. The overall model was highly statistically significant (F(6,82) = 156.12; *p* < 0.0000). A detailed analysis of coefficients (Table 6) and their graphical representation (Figure 1) revealed three statistically significant predictors of quality of life in this group: CDI-2™ subscale D (Interpersonal Problems), the strongest negative predictor (b* = −2.43; *p* < 0.000001); CDI-2™ subscale C (Ineffectiveness), the strongest positive predictor (b* = 2.26; *p* < 0.000001); and CDI-2™ subscale A (Negative Mood/Somatic Symptoms) (b* = 1.08; *p* = 0.000392).

The CDI-2™ subscale B (Low Self-Esteem) did not show a statistically significant relationship with quality of life (*p* = 0.603), while age (*p* = 0.060) and self-assessed health status (*p* = 0.082) reached the level of statistical tendency.

**Figure 2:**
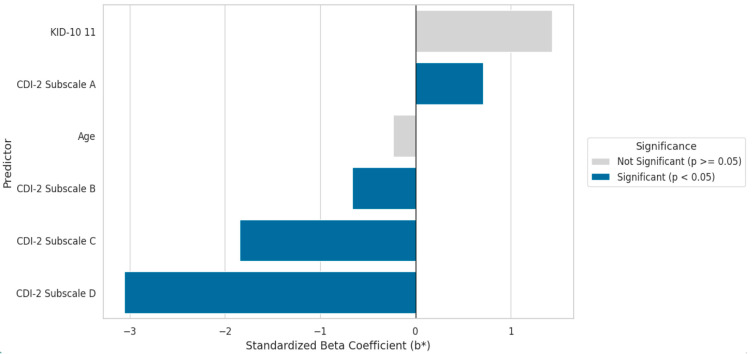
Predictors of Quality of Life—Older Cohort (N = 76).

For the Older Group, the model demonstrated a good fit to the data (R = 0.912) and accounted for 81.7% of the variance in quality of life (adjusted R^2^ = 0.8173). The model was highly statistically significant (F(6,69) = 56.904; *p* < 0.0000). In this model (Table 7, Figure 2), four statistically significant predictors were identified. Similar to the Younger Group, the key predictors with the greatest influence were CDI-2™ subscale D (Interpersonal Problems, b* = −3.05; *p* < 0.000001) and subscale C (Ineffectiveness, b* = −1.85; *p* < 0.000001). Subscale A (Negative Mood/Somatic Symptoms) was also statistically significant (b* = 0.71; *p* = 0.034).

However, the crucial difference compared to the Younger Group was the significance of CDI-2™ subscale B (Low Self-Esteem), which in the older group became a significant negative predictor of quality of life (b* = −0.67; *p* = 0.038). Age and self-assessed health status were completely non-significant in this model.

### 3.5. Comparison of Predictors Between Younger and Older Cohorts

Comparison of the two regression models indicates that although both demonstrate very strong predictive capacity, the model for the Younger Cohort (explaining 91.4% of variance) shows slightly higher explanatory power than the model for the Older Cohort (81.7% of variance). In both age groups, the universal and key predictors of quality of life were CDI-2™ subscales A (Negative Mood), C (Ineffectiveness), and D (Negative Self-Esteem). The most significant difference between the models concerns the role of CDI-2™ subscale B (Interpersonal Problems). It was statistically non-significant for younger children (*p* = 0.603), whereas it became a significant negative predictor in the older group (*p* = 0.038). This statistical difference points to a developmental shift, suggesting that interpersonal difficulties gain significance as a determinant of subjective quality of life during late adolescence.

## 4. Discussion

The findings of the present study reveal a distinct dichotomy in Health-Related Quality of Life (HRQoL). Rather than a uniform distribution, we observed two contrasting subgroups: a substantial minority (approximately one-third) experiencing critically low well-being, coexisting with a majority of survivors who report relatively high functioning. This pattern identifies a specific, clinically vulnerable subgroup whose risk profile is moderated by intrapsychic factors and developmental context. The results are consistent with international research that increasingly challenges the paradigm of uniform and successful psychosocial adjustment following the completion of oncological treatment during adolescence. These data confirm the existence of a clinically sensitive subgroup of survivors whose risk profile is moderated by intrapsychic factors and developmental context.

### 4.1. Polarization of HRQoL and Prevalence of Psychosocial Burdens

A key finding of the present study is the strong polarization of perceived quality of life, with nearly one-third of the surveyed adolescents falling into the critically low well-being range. Although alarming, this result aligns with data showing considerable psychosocial burden within this population. In the Canadian YACPRIME study (n = 195 survivors vs. n = 665 controls), Schulte et al. (2021) reported a similar proportion (31.8%) of survivors describing poor physical health and an even higher percentage (49.7%) reporting poor mental health. In that study, survivors evaluated approximately 6.48 years post-treatment, exhibited markedly lower HRQoL scores—both physical (F[1,818] = 52.80) and mental (F[1,818] = 83.54), compared with the control group [13].

Other studies confirm a high frequency of individual symptom occurrence. In a large Canadian survey (n = 575 survivors aged 18–34 years), Jones et al. (2020) found that 90% of participants reported at least one emotional concern (mean = 3.77) and 88% reported at least one physical concern (mean = 3.98). The most frequently reported problems were fear of recurrence (83%), fatigue (78%), and depression (66%) [14].

However, the overall picture remains complex. A systematic review of 36 European studies (n = 14,342 survivors) conducted by Larsen et al. (2023) showed that although most studies with comparison groups (23 of 31 (only 31 studies reported a comparison group)) reported lower quality of life (QoL) among survivors, the observed effect sizes were generally small to moderate. Interestingly, five studies included in the review found even higher QoL among survivors, mainly in domains related to mental health and vitality [15].

Contrastingly, while some large population-based studies, such as Han et al. (2021), report HRQoL scores comparable to norms, these findings are heavily skewed by older adult survivors (approx. 60% aged ≥65 years) [16]. Such data aggregation tends to mask the specific developmental challenges of adolescence. As emphasized by Janssen et al. (2021), AYAs represent a distinct population with unique survivorship needs that differ significantly from both pediatric and older adult cohorts. Consequently, our focus on a strictly adolescent cohort exposes specific vulnerabilities—such as the identified dichotomy in well-being—that remain invisible in broader, age-heterogeneous studies [16,17].

In a broader global context, our findings align with the diverse challenges faced by healthcare systems worldwide. As demonstrated by a multinational study by Tso et al. (2025) across 28 Asian countries/regions, the availability of specialized long-term follow-up (LTFU) care is strongly correlated with economic resources, with less than half of institutions having dedicated survivor clinics. Similarly, in Poland, despite high survival rates, the psychosocial support system is still developing. Culturally, Polish survivors may exhibit adaptive mechanisms similar to those described in the literature as post-traumatic growth (PTG). Janssen et al. (2021) note that despite high levels of distress, many AYA survivors experience positive psychological changes, such as resilience and a greater appreciation for life. This may explain the ‘self-esteem paradox’ observed in our cohort—high levels of negative mood coexisting with preserved self-worth [17,18,19].

### 4.2. Psychological Profile: The Self-Esteem Paradox and the Role of Hope

The study revealed a distinctive psychological profile characterized by high levels of negative mood and feelings of inefficacy, yet not accompanied by universally low self-esteem. However, a key finding was the paradox that low self-esteem (measured by the CDI-2™ subscale D), despite its relatively low mean across the group, emerged as the strongest negative predictor of overall quality of life.

In a Dutch study by Langeveld et al. (2004), involving a cohort of young adults (aged 18–30 years; n = 400 survivors vs. n = 560 controls), similar patterns were observed. Despite the developmental difference compared to our adolescent sample (11–18 years), they likewise found no significant differences in mean self-esteem scores between survivors and controls. Yet, in their multiple regression model, low self-esteem proved to be one of the strongest predictors of poorer quality of life across all dimensions. This continuity of findings suggests that the protective function of self-esteem is a stable mechanism that persists from adolescence into young adulthood [18].

This relationship has also been confirmed in Asian research. In a study of 176 survivors aged 10–16 years in Hong Kong, Ho et al. (2019) found that the level of hope (measured with the HHI) was the strongest predictor of HRQoL (β = 0.32), followed closely by depressive symptoms (CES-DC; β = −0.24) and self-esteem (RSES; β = 0.22). This suggests that self-esteem forms part of a broader adaptive [20].

Similarly, in a Taiwanese study (n = 98 survivors), Chou and Hunter (2009), using Haase’s Adolescent Resilience Model (ARM), reported that QoL was most strongly and negatively correlated with individual risk (r = −0.67). In their qualitative analysis, this risk was defined as Loss of Self, encompassing loss of self-efficacy, loss of self-esteem, and loss of hope for the future. Thus, although low self-esteem is not a universal issue, in adolescents where it occurs, it functions as a central destructive factor for overall well-being [21].

The observed pattern “I feel bad, but I am not bad” strongly suggests the presence of active adaptive mechanisms, potentially manifesting as Post-Traumatic Growth (PTG), a phenomenon well described in oncological literature. In this context, the findings may be interpreted as the coexistence of distress (the “cost” of illness) alongside a preserved sense of self-worth (the “gain” derived from successfully navigating the crisis) [17].

### 4.3. Developmental Context and Interpersonal Functioning

A clinically significant finding of the present study is the identification of a developmental shift: interpersonal problems (measured by CDI-2™ subscale B) became a statistically significant predictor of quality of life only in the older adolescent group (Group S). This finding is fully consistent with developmental theory, which emphasizes the central role of peer relationships during adolescence.

Perez et al. (2020), in their review, describe the period immediately following treatment as a “sensitive re-entry period.” The diagnosis and treatment process severely disrupts key developmental tasks characteristic of this life stage, such as building autonomy, establishing peer and romantic relationships, pursuing education, and initiating career development [22].

A Danish qualitative study by Ingersgaard et al. (2021) on the RESPECT intervention, in which healthy peers (“ambassadors”) accompanied patients during hospitalization, provides deep insight into this dynamic. Survivors described their ambassadors as crucial liaison persons and promoters of normalcy and identity continuity. Most importantly, ambassadors became “behind-the-scenes friends” who, unlike other peers, developed a profound, shared understanding of the illness experience by witnessing its effects firsthand. However, this study also revealed the emotional cost of such insight. Adolescents reported feelings of vulnerability and inferiority when peers observed their extreme weakness and treatment side effects (e.g., vomiting). One participant described this as “the price of their understanding” [23].

The quantitative data obtained in the present study, showing the increasing impact of interpersonal problems on HRQoL in the older group, reinforce the notion that success or failure in this crucial process of peer reintegration becomes a central determinant of overall psychological well-being in late adolescence.

### 4.4. Financial Toxicity and Socioeconomic Status as Hidden Determinants of Health

When analyzing predictors of reduced well-being, the present study, similar to many others in this field, focused primarily on clinical and internal variables (e.g., depressive symptoms). However, a growing body of evidence indicates that external socioeconomic factors, often described as “financial toxicity,” play a crucial, if not dominant, role in determining the quality of life among adolescent cancer survivors.

The IGHG (International Guideline Harmonization Group) review by Marchak et al. (2022) clearly classifies unemployment (evidence level A/B), lower educational attainment (A/B), and low household income (B) as major risk factors for a wide spectrum of mental health disorders, including depression, anxiety, PTSD, and psychological distress [22,24].

The magnitude of this relationship was strikingly quantified in a Canadian study by Schulte et al. (2021). In a multiple regression model, an annual household income below $40,000 emerged as the strongest single predictor of poor physical health (AOR = 8.32; 95% CI) and lower mental health component scores (GMH; β = 3.44). These consequences are particularly severe for adolescents, who at the time of diagnosis lack the financial stability available to older patients [13].

Perez et al. (2020) emphasize that this age group faces unique challenges, such as interrupted education, inability to obtain first employment, student debt, and out-of-pocket expenses for procedures (e.g., oncofertility treatments) that are often not reimbursed. In the AYA HOPE study, financial counseling was reported as the most frequently unmet need among participants [22].

Given the present study’s findings of a high prevalence of depressive symptoms, it is critically important to determine whether this distress reflects a reaction to objective, material life difficulties rather than solely an intrapsychological or neurohormonal consequence of the illness. This implies that future interventions aimed at improving HRQoL cannot be limited solely to psychotherapy. Instead, they must adopt a biopsychosocial model, integrating support in career and educational counseling, as well as financial navigation, to address the root causes of socioeconomic distress.

The ‘sense of ineffectiveness’ (CDI-2 scale C) identified in the study should not be treated merely as a subjective depressive symptom, but as a signal of real risk for functional difficulties. This is consistent with the latest guidelines from the International Late Effects of Childhood Cancer Guideline Harmonization Group (IGHG) developed by Devine et al. (2022). This review confirms that survivors are at risk for lower educational achievement and unemployment, with psychological distress being a significant risk factor for these adverse outcomes. Consequently, routine surveillance of educational and employment progress is recommended for all survivors, which, in light of our findings, should become a standard of care in Polish centers [17].

### 4.5. Neurocognitive Deficits as a Potential Source of Functional Impairment

In the present study, the CDI-2™ questionnaire identified “Lack of Effectiveness” as one of the key problematic dimensions. In standard clinical interpretation, this symptom is often understood as a component of the depressive triad alongside negative mood and low self-esteem. However, in the population of post-oncological survivors, particularly those exposed to therapies targeting the central nervous system (CNS), this finding may conceal a distinct and highly prevalent issue: late-onset neurocognitive sequelae.

The systematic review by Michel et al. (2020) reports that neurocognitive problems are “highly prevalent” and may affect more than 35% of survivors. Importantly, these deficits do not merely involve a global reduction in IQ but rather manifest as specific impairments in processing speed, executive functioning (verbal fluency, cognitive flexibility), memory, and attention. Although the greatest risk is observed among patients treated with CNS radiotherapy, studies have shown that even modern chemotherapy-only protocols for acute lymphoblastic leukemia (ALL) are associated with an increased risk of attention and executive dysfunction [25].

These deficits have a direct impact on adolescents’ daily functioning. Michel et al. (2020) cite a meta-analysis showing that survivors are significantly less likely to complete secondary or higher education and more likely to require academic support [25]. In Jones et al. (2020), “changes in concentration or memory” were the third most frequently reported physical problem (49%) and strikingly had the highest rate of unmet need (47% of respondents reported difficulty obtaining help) [14].

What was measured in the present study as a subjective “sense of inefficiency” may, in reality, reflect an objective difficulty in meeting academic demands, resulting from slower information processing or challenges in task organization—rather than solely reduced motivation or mood. This has fundamental implications for future research and clinical practice. It suggests that interventions relying solely on cognitive-behavioral therapy (CBT) for depression may be insufficient. This is supported by a meta-analysis by Zhang et al. (2021), which found that existing interventions targeting academic outcomes produced negligible and statistically non-significant effects (d = 0.257, *p* = 0.0508). Future research should therefore focus on validating and implementing specific remedial interventions, such as pharmacotherapy (e.g., methylphenidate) or computer-based cognitive training (e.g., Cogmed), both of which have shown promising results in pilot studies aimed at improving working memory and attention among survivors [26].

### 4.6. Study Limitations

When interpreting the obtained results, several methodological limitations must be taken into account. As in many studies in this field, the present research employed a cross-sectional design. This approach precludes the establishment of causal relationships. It is therefore impossible to determine conclusively whether depressive symptoms lead to decreased HRQoL and social functioning, or whether primary functional difficulties (e.g., resulting from treatment-related complications) secondarily generate depressive symptoms. Only longitudinal studies can trace trajectories and identify mutual influences over time.

Another limitation concerns the sampling method. Recruitment was conducted in four leading oncology centers, representing a convenience sample rather than a population-based one. This entails a risk of selection bias. Clinical recruitment might have attracted individuals with greater psychosocial difficulties, or conversely, might have excluded those experiencing the most severe problems—both physical and psychological—who have dropped out of long-term outpatient follow-up. While convenience sampling limits generalizability to the entire survivor population, this recruitment strategy was chosen for specific logistical and methodological reasons. Survivors who have disengaged from long-term follow-up often exhibit medical avoidance or lack of insight into their health needs. Consequently, this subgroup is historically non-responsive to remote survey invitations (e.g., mail or email). By recruiting within the clinic, we ensured a high response rate and data completeness, which would likely be unattainable in a population-based design without significant attrition bias. Furthermore, the clinical setting allowed for immediate psychological support if the questionnaire triggered distress, an ethical safeguard unavailable in remote surveying.

The rigorous exclusion criteria applied (e.g., ongoing treatment, severe somatic complications, active psychiatric hospitalization) were necessary to ensure sample homogeneity and ethical conduct; however, they strictly limited the analysis to relatively high-functioning survivors. Consequently, it is impossible to statistically assess the excluded subgroup within our dataset. Therefore, our results should be interpreted as a conservative estimate. It is highly probable that the true prevalence of psychosocial dysfunction in the total population of survivors—including those with severe sequelae who were systematically excluded—is significantly higher than the 32.7% observed in this study.

The study relied solely on self-report instruments (KIDSCREEN-10, CDI-2™). While both are psychometrically validated tools, they do not provide objective measures of functioning. Diagnostic heterogeneity within the sample, although reflective of real clinical conditions, complicates the drawing of conclusions specific to certain cancer types—an important consideration given that survivors of CNS tumors or HSCT recipients are known high-risk groups.

The heterogeneity of the adolescent age range also represents a recognized methodological challenge. Although the study sample (ages 11–18) is relatively narrow, it encompasses the critical transition from early to late adolescence, which may mask more subtle developmental differences in needs and predictors of distress.

Finally, the present study contributes valuable data from Central and Eastern Europe. As noted by Larsen et al. (2023) in their systematic review of European studies, “no studies from Eastern Europe were identified in the literature search,” which limits the generalizability of previous findings. Our Polish data help to fill this important geographical gap [15].

### 4.7. Practical Implications and Directions for Future Research

Despite the outlined methodological limitations, the findings of this study have significant implications for the organization of survivorship care and provide clear directions for future research, particularly within the context of the underdeveloped support systems in Poland.

The most important clinical implication is the urgent need to implement systematic and universal mental health surveillance as an integral component of survivorship care. This recommendation is fully consistent with international IGHG guidelines. Both the Mental Health Working Group (Marchak et al., 2022) and the Fatigue Working Group (Christen et al., 2020) issued strong recommendations for conducting distress and fatigue screenings for all survivors during every follow-up visit [24,27]. The present study confirms that the Polish adolescent survivor population is no exception and urgently requires implementation of these standards.

Our findings provide empirical evidence for the existence of a “short-term care gap.” De Beijer et al. (2023), in developing the PanCare guidelines, identified a critical lack of evidence-based recommendations for follow-up during the early survivorship period (defined as 0–5 years post-diagnosis) [28]. The present study, focusing precisely on adolescents within this time window, reveals a high prevalence of psychological problems and confirms that this period is crucial for introducing early interventions before symptoms become chronic.

Perhaps the most alarming finding concerns the mismatch between existing interventions and the specific needs of adolescents. The study demonstrates that this group represents a high-risk population, yet, according to the large meta-analysis by Zhang et al. (2021) (61 studies, n = 4402), psychosocial and behavioral interventions are significantly less effective among adolescents and young adults (AYAs) (d = 0.211) than among pediatric patients (d = 0.573). Moreover, interventions targeting social outcomes, the primary problem identified in our older cohort, had only a small effect size (d = 0.219). This means that, as healthcare systems, we are currently offering the weakest tools to those who need them the most [26].

In light of these findings, future research must prioritize the development and rigorous evaluation of age-appropriate, developmentally tailored interventions for adolescents. It is necessary to move away from pediatric models toward programs addressing the specific challenges of this age group, peer reintegration, fear of cancer recurrence (FCR), body image issues, and support in coping with “taboo” topics such as sexuality, fertility, and financial insecurity, as emphasized by Perez et al. (2020). Research should explore innovative formats, including internet-based or mobile interventions. However, caution is warranted: the meta-analysis by Wang et al. (2020) showed that while online programs effectively reduce depression (SMD = −0.58) and fatigue (MD = −9.83), their impact on overall quality of life (QoL) is statistically non-significant (*p* = 0.70). Finally, it is essential to conduct longitudinal studies, which, unlike our cross-sectional design, would enable researchers to track symptom trajectories and identify optimal time windows for early intervention [29].

Implementing systematic surveillance requires proven tools. Experiences from neighboring healthcare systems, such as those in Germany and Austria, described by Gebauer et al. (2023), point to the effectiveness of the ‘Survivorship Passport’ and risk-stratified care in implementing IGHG guidelines. Furthermore, the study by Tso et al. (2025) demonstrated that institutions adhering to formalized guidelines are significantly more likely to provide comprehensive health risk counseling and multidisciplinary assessment. This suggests that adapting international standards (e.g., IGHG) to the Polish context is a crucial step towards bridging the care gap for adolescent survivors [4].

## 5. Conclusions

The psychological profile of the studied cohort appears highly specific. Intense emotional distress, manifested as negative mood and a sense of inefficacy, does not necessarily lead to the internalization of a negative self-image, suggesting a distinctive coping mechanism. Quality of life within this sample is not a uniform phenomenon but rather strongly polarized, indicating the existence of two distinct subgroups within the population: one functioning relatively well and another, comprising approximately one-third of participants, falling within the clinical risk zone.

The findings further demonstrate that the key determinant of QoL is not, as might be assumed, the general mood level, but rather the strength of negative self-evaluation. Despite its relatively low mean across the group, self-esteem emerged as the most powerful predictor of overall well-being. Regression model analyses also revealed a clear developmental shift in the factors shaping well-being. While intrapsychic components such as self-esteem, mood, and perceived effectiveness act as universal predictors, the role of interpersonal difficulties becomes significant only in the older adolescent group.

From a clinical perspective, these results imply the necessity of differentiated intervention strategies. Across all age groups, therapeutic work focused on self-esteem enhancement appears to be central. However, in older adolescents, effective support must also incorporate the social context, particularly peer relations and interpersonal functioning, which constitute crucial determinants of psychosocial adaptation during this developmental stage.

## Figures and Tables

**Table 1:** Demographic and Environmental Characteristics of the Sample (N = 165).

Variable	Category	N	%
Education level	Primary School	89	53.94
	Secondary School	76	46.06
Gender	Male	87	52.73
	Female	78	47.27
Age	11	12	7.27
	12	31	18.79
	13	35	21.21
	14	11	6.67
	15	6	3.64
	16	19	11.52
	17	18	10.91
	18	33	20.00
Family situation	Two-parent family	132	77.65
	Parents divorced/separated	17	10.00
Family situation	One parent deceased	16	9.41
Siblings	None	27	16.34
	One sibling	83	50.30
	Two or more siblings	55	33.33
Living arrangement	With both parents	133	80.61
	Other (with one parent, stepparent, or in a dormitory)	32	19.39
Mother’s education	Higher	76	46.06
	Secondary	66	40.00
	Vocational/unknown	23	13.94
Father’s education	Higher	67	40.61
	Secondary	65	39.39
	Vocational/unknown	33	20.00
Primary school grade	Grade 6	33	20.00
	Grade 7	46	27.88
	Grade 8	10	6.06
Primary school grade	Grade 1	8	4.85
	Grade 2	32	19.39
	Grade 3	30	18.18
	Grade 4	6	3.64

**Table 2:** Descriptive Statistics for the KIDSCREEN-10 and CDI-2™ Scales (N = 165).

Scale	N	Min–Max	M	SD	Mdn	D
KIDSCREEN-10—Total Score	165	22–49	35.93	5.17	37	38 and 39

M—Mean; SD—Standard Deviation; Mdn—Median; D—Mode; Min–Max—Minimum and Maximum values.

**Table 3:** Frequency Distribution of KIDSCREEN-10 Scores According to Clinical Risk Categories for the Polish Population (N = 165).

Interpretative Category	N	Frequency [%]
1. ≥Mean (High Well-Being)	73	44.24
2. ≥Mean − 1 SD	14	8.48
3. ≥Mean − 2 SD	24	14.55
4. <Mean − 2 SD (Low/Critical Well-Being)	54	32.73

SD—Standard Deviation.

**Table 4:** Descriptive Statistics for Depressive Symptoms (CDI-2™) (N = 165).

Scale	N	Min–Max	M	SD	Mdn	D
CDI-2™—Total Score	165	18–60	56.06	4.2	56	56
CDI-2™ Symptom Subscales						
Subscale A—Negative Mood	165	17–22	19.93	1.55	20	21
Subscale B—Interpersonal Problems	165	11–16	13.26	1.27	13	14
Subscale C—Ineffectiveness	165	13–17	15.45	1.25	16	16
Subscale D—Negative Self-Esteem	165	5–12	8.02	2.5	7	5

M—Mean; SD—Standard Deviation; Mdn—Median; D—Mode; Min–Max—Minimum and Maximum values.

**Table 5:** Spearman’s Rank Correlation Matrix for the Total Sample (N = 165).

Variable	Age	KIDSCREEN-10 Self-Rated Health	KIDSCREEN-10 Total Score	CDI-2 Subscale A	CDI-2 Subscale B	CDI-2 Subscale C	CDI-2 Subscale D
Age	1	**0.31**	**−0.27**	**−0.31**	−0.03	−0.04	0.22
KIDSCREEN-10 Self-rated Health	**0.31**	1	**0.64**	**−0.29**	**−0.39**	0.02	−0.51
KIDSCREEN-10 Total Score	**−0.27**	**0.64**	1	**0.69**	**0.49**	0.04	**−0.89**
CDI-2™ Subscale A	**−0.31**	**−0.29**	**0.69**	1	**0.40**	−0.05	**−0.70**
CDI-2™ Subscale B	−0.03	**−0.39**	**0.49**	**0.40**	1	0.06	**−0.62**
CDI-2™ Subscale C	−0.04	0.02	0.04	−0.05	0.06	1	−0.03
CDI-2™ Subscale D	0.22	**−0.51**	**−0.89**	**−0.70**	**−0.62**	−0.03	1
CDI-2™ Emotional Problems	−0.19	**−0.38**	**0.71**	**0.87**	**0.80**	−0.01	**−0.73**
CDI-2™ Functional Problems	0.15	**−0.46**	**0.30**	0.24	0.25	**0.55**	**−0.58**
Age at Diagnosis	**0.57**	**0.36**	−0.09	0.13	0.15	0.02	0.11
Years Since Treatment	−0.19	0.01	−0.01	0.05	**−0.28**	−0.05	0.06

Bold values indicate statistical significance at *p* < 0.05. Coefficients are presented as Spearman’s rho (rs).

**Table 6:** Summary of Regression Results for Younger Cohort (Dependent Variable: KIDSCREEN-10 Total Score; N = 89). Bold values indicate statistical significance at *p* < 0.05.

Variable	b (Unstandardized)	b* (Standardized)	t (82)	*p*-Value
Intercept	5.32	-	0.46	0.645
Age	−0.08	−0.66	−1.91	0.060
KID-10 Item 11 (Self-rated Health)	−0.07	−0.72	−1.76	0.082
CDI-2 Subscale A (Negative Mood/Physical Symptoms)	0.24	1.08	3.70	**<0.001**
CDI-2 Subscale B (Interpersonal Problems)	0.02	0.12	0.52	0.603
CDI-2 Subscale C (Ineffectiveness)	0.42	2.26	10.53	**<0.001**
CDI-2 Subscale D (Negative Self-Esteem)	−0.81	−2.43	−12.99	**<0.001**

**Table 7:** Summary of Regression Results for Older Cohort (Dependent Variable: KIDSCREEN-10 Total Score; N = 76). Bold values indicate statistical significance at *p* < 0.05.

Variable	b (Unstandardized)	b* (Standardized)	t (69)	*p*-Value
Intercept	79.36		6.00	**<0.001**
Age	−0.04	−0.24	−0.70	0.486
KID-10 Item 11 (Self-rated Health)	0.20	1.43	1.50	0.137
CDI-2 Subscale A (Negative Mood/Physical Symptoms)	0.16	0.71	2.16	**0.034**
CDI-2 Subscale B (Interpersonal Problems)	−0.16	−0.67	−2.12	**0.038**
CDI-2 Subscale C (Ineffectiveness)	0.43	−1.85	5.89	**<0.001**
CDI-2 Subscale D (Negative Self-Esteem)	−1.14	−3.05	−6.87	**<0.001**

## Data Availability

The data presented in this study are available on request from the corresponding author due to ethical and privacy reasons.
